# Halophytes: Potential Resources for Salt Stress Tolerance Genes and Promoters

**DOI:** 10.3389/fpls.2017.00829

**Published:** 2017-05-18

**Authors:** Avinash Mishra, Bhakti Tanna

**Affiliations:** ^1^Marine Biotechnology and Ecology Division, Central Salt and Marine Chemicals Research Institute (CSIR)Bhavnagar, India; ^2^Academy of Scientific and Innovative Research, Council of Scientific and Industrial ResearchNew Delhi, India

**Keywords:** abiotic stress, halophytes, promoter, salinity, salt responsive genes, salt stress, stress tolerance, transgenic

## Abstract

Halophytes have demonstrated their capability to thrive under extremely saline conditions and thus considered as one of the best germplasm for saline agriculture. Salinity is a worldwide problem, and the salt-affected areas are increasing day-by-day because of scanty rainfall, poor irrigation system, salt ingression, water contamination, and other environmental factors. The salinity stress tolerance mechanism is a very complex phenomenon, and some pathways are coordinately linked for imparting salinity tolerance. Though a number of salt responsive genes have been reported from the halophytes, there is always a quest for promising stress-responsive genes that can modulate plant physiology according to the salt stress. Halophytes such as *Aeluropus, Mesembryanthemum, Suaeda, Atriplex, Thellungiella, Cakile*, and *Salicornia* serve as a potential candidate for the salt-responsive genes and promoters. Several known genes like antiporters (*NHX, SOS, HKT, VTPase*), ion channels (Cl^−^, Ca^2+^, aquaporins), antioxidant encoding genes (*APX, CAT, GST, BADH, SOD*) and some novel genes such as *USP, SDR1, SRP* etc. were isolated from halophytes and explored for developing stress tolerance in the crop plants (glycophytes). It is evidenced that stress triggers salt sensors that lead to the activation of stress tolerance mechanisms which involve multiple signaling proteins, up- or down-regulation of several genes, and finally the distinctive or collective effects of stress-responsive genes. In this review, halophytes are discussed as an excellent platform for salt responsive genes which can be utilized for developing salinity tolerance in crop plants through genetic engineering.

## Introduction

Salinization is a worldwide problem in which salts gradually accumulate in the soil. In this process, water-soluble salts are deposited in the soil to an extent that affects crop productivity, microbial community, and agricultural economics (FAO, 2016). The salinization eventually transforms a fertile land to barren. The process destroys all vegetation and other organisms living in the soil and thus it is detrimental to the environmental health. Most of the world's land is not cultivated, and over 2% of the total land is affected by salinity (FAO; Land and Plant Nutrition Management Service). A significant proportion of cultivated land is salt-affected, and out of the current 230 million ha of irrigated land, 45 million ha are salt-affected whereas 32 million are salt-affected to varying degrees (FAO, 2008).

Halophytes are salt-resistant or salt-tolerant plants and have remarkable ability to complete their life cycle in saline condition. During evolution, they have developed different morphological, anatomical, and physiological strategies to proliferate in high-salt environments (Flowers and Colmer, 2008; Grigore et al., 2014). Halophytes have occasionally been reviewed for their general physiology (Flowers, 1985), ecophysiology (Ball, 1988), photosynthesis (Rozema and Van Diggelen, 1991; Lovelock and Ball, 2002), response to oxidative stress (Jithesh et al., 2006), flooding tolerance (Colmer and Flowers, 2008), salinity tolerance (Flowers and Colmer, 2008), and adaptations (Flowers et al., 2015). Additionally, other researchers have also examined halophytes under special topics as sustainable cultivation, saline agriculture, and integrative anatomy (Rozema et al., 2013; Grigore et al., 2014; Flowers et al., 2015; Xu et al., 2016).

Halophytes that consistently require salt for their growth are referred to “obligate halophytes” (Braun-Blanquet, 1932), but some halophytes have the ability to grow on the soil devoid of salt are called as “facultative halophytes” (Polunin, 1960). Halophytes are obligate and facultative based upon salt demand and tolerance for sodium salts. Previously, a study has been carried out with some selected halophytes to investigate the salt requirement for growth and development (Grigore et al., 2012). Researchers concluded that salts are not compulsorily required for the development of halophytic species but the availability of water and nutrients are also key limiting factors for growth in natural saline habitats (Grigore et al., 2012). Further, they hypothesized that halophytes are mostly distributed in saline areas to avoid competition with glycophytic species.

Ecophysiological characteristics are used by Cushman (2001) to differentiate between obligate, facultative, and habitat-indifferent halophytes. Habitat-indifferent halophytes are undistinguished to their habitat, usually, prefer to live in a salt-free soil but have the ability to cope with the saline condition (Cushman, 2001). Recently, Grigore and Toma (2010) proposed a new type of classification of halophytes; extreme-halophyte (irreversible and reversible) and meso-halophytes, by integrating anatomy observations with ecological factors (salinity). Extreme-halophytes are well-adapted extreme halophytes and growing exclusively in saline environments. Furthermore, the habitat of these halophytes may be irreversible or reversible. They concluded that Chenopodiaceae (now included in the family Amaranthaceae) succulent species (*Salicornia, Suaeda, Halimione*, and *Petrosimonia*) are extreme halophytes and best adapted to high salinity conditions. Halophytes such as *Atriplex, Bassia*, and *Camphorosma* are not strictly related to increased salinity, therefore may be classified as reversible halophytes. There is always a difficulty with the distinct terminology of halophytes because the definition is still obscure and Grigore et al. (2010) discussed a short historical evolution of halophytes definition in chronological order.

Advanced and novel stress-tolerant mechanisms are difficult to study with the model plant *Arabidopsis* as some mechanisms are unique to halophytes. The comparative genomics of *Mesembryanthemum crystallinum* and *Arabidopsis thaliana* confirmed that some transcripts present in former and later do not have counterparts (Wang et al., 2004). Some other halophytes, *Suaeda* species, and *Atriplex* species have been investigated to unravel molecular mechanism of stress tolerance. Among all, *Thellungiella halophila* is one of the halophytes emerging as a model halophyte for the study of abiotic stress tolerance mechanism (Wang et al., 2004; Amtmann, 2009). Halophyte *Cakile maritima* and *Suaeda maritima* (Megdiche et al., 2009; Sahu and Shaw, 2009) are considered as model plants for the transcript profiling and *Salicornia brachiata* as a potential halophyte for new and useful salt-tolerant genes (Singh et al., 2016; Udawat et al., 2016, 2017). In this review, halophytes are discussed as resources for salt stress tolerance genes, which can be explored further for developing abiotic stress tolerance crops for sustainable agriculture.

### Salt tolerance mechanism in halophytes: a glimpse

Halophytes are well-adapted and thrive under high salinity by using two strategies, salt tolerance, and salt avoidance. Generally, halophytes follow three mechanisms of salt tolerance; reduction of the Na^+^ influx, compartmentalization, and excretion of sodium ions (Flowers and Colmer, 2008, 2015). Adaptations involved in salt avoidance are secretion, shedding, and succulence (discussed in Waisel, 1972; Rozema, 1995; Aslam et al., 2011; Shabala et al., 2014). In brief, secretion is a complex mechanism, and salt-secreting structures (salt hairs or salt glands) are distributed in halophytes. Some halophytes are capable of excreting excess salt in the form of a liquid which becomes crystals in contact with air and may visible on the plant leaf surface. In some halophytes, shedding of the old leaves which are grown under high salt concentrations is another strategy to avoid the salt toxicity. Grigore et al. (2014) discussed the different aspects of the various adaptive structures of halophytes in an integrative way at the anatomy level.

**Figure 1:**
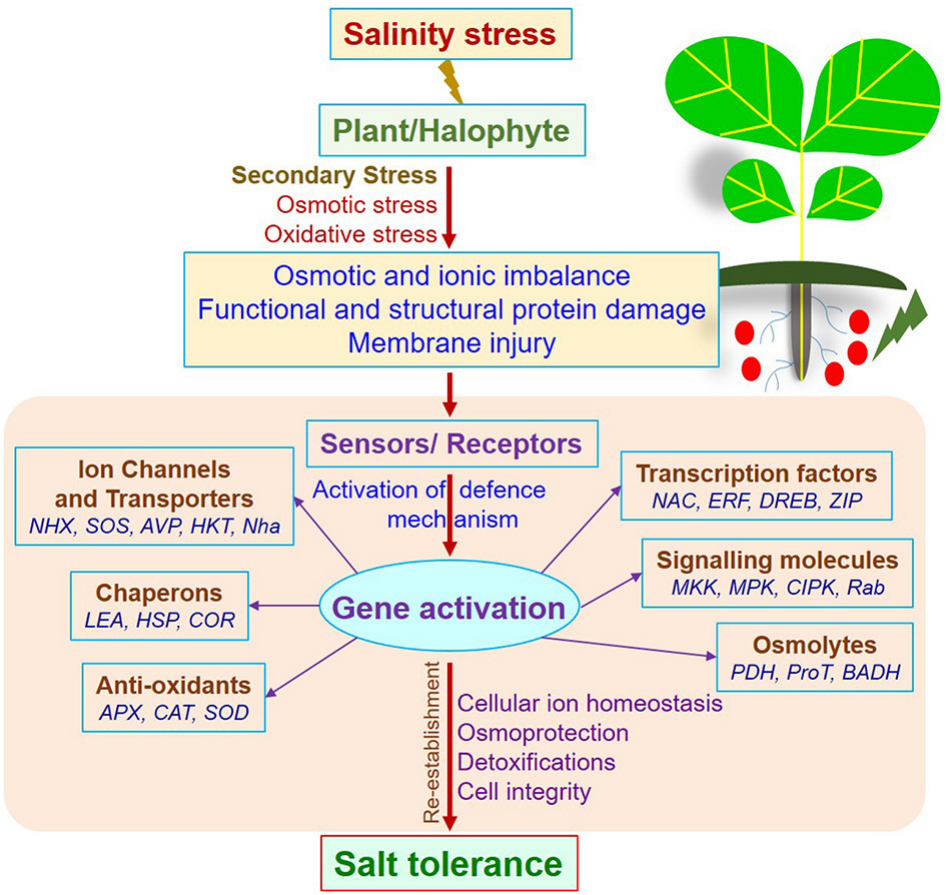
**A generalized schematic representation of salinity stress tolerance mechanism in a plant**.

The salt tolerance mechanism is coordinately linked (Figure 1) with signal transduction, ROS generation and detoxification pathways, osmoregulation or ion homeostasis through osmoprotectants, and differential expression of salt responsive genes and transcription factors (Flowers and Colmer, 2008; Rajalakshmi and Parida, 2012; Himabindu et al., 2016; Khan et al., 2016; Muchate et al., 2016). ROS detoxification pathways include antioxidative enzymes which play a protective role in scavenging toxic radicals (Das and Strasser, 2013). Salt sequestration into cell vacuoles through transporters is another key mechanism employed by halophytes to maintain a high cytosolic K^+^/Na^+^ ratio and thus control the salt concentrations in the cytosol (Kronzucker and Britto, 2011; Sreeshan et al., 2014). Accumulation of osmoprotectants such as proline, glycine betaine, polyphenols, soluble sugars, and inorganic ions is a conventional plant defense mechanism routinely used by halophytes to cope with stresses (Lokhande and Suprasanna, 2012; Patel et al., 2016). At the molecular level, halophytes impart salt tolerance by regulating stress-responsive genes through ABA-dependent or ABA-independent regulation mechanism. Overall, salt tolerance in a halophyte is a complex network that involves the interactions of multiple physiological responses directive by several genes and gene products (Figure 1). Overall, halophytic salt tolerance defense mechanism includes changes in ion homeostasis (both influx and efflux), the formation of osmoprotectants, activation of crosstalk genes, induction of antioxidants, and the development of salt gland or bladders (Shabala et al., 2014; Slama et al., 2015; Himabindu et al., 2016).

### Salt responsive genes from halophytes: an overview

Halophytes have been studied extensively for their ecological, physiological, anatomical, and biochemical responses toward salinity (Flowers and Colmer, 2008; Aslam et al., 2011; Shabala, 2013; Ventura et al., 2015). Furthermore, halophytes were also explored for saline agriculture and examined as bioenergy crop (Rozema and Schat, 2013; Sharma et al., 2016). However, little information is available on well-defined molecular defense mechanism of halophytes against salt stress (Anjum et al., 2012; Joshi et al., 2015). Surprisingly, a non-tolerant plant, *A. thaliana* is widely explored as a model plant to investigate the molecular mechanism of salt stress tolerance (Sanders, 2000; Zhu, 2001). Additionally, this plant is also exploited for the gene mining of salt stress-responsive genes for the improvement of tolerance in transgenic crops (Zhu, 2000).

It is a general assumption that halophytes are salt resistant while glycophytes are sensitive, but there are several species considered traditionally as glycophytes are resistant or tolerant to salt and some halophytes may be sensitive to several environmental stresses. Recently, it is experimentally proven that halophytes are one of the most appropriate models for the studying different salt stress tolerance mechanisms (Shabala, 2013; Flowers and Colmer, 2015; Himabindu et al., 2016). A number of evidences suggest that all plants have almost similar salt tolerance regulatory mechanisms and there are quantitative differences rather than qualitative between halophyte and glycophyte (Anjum et al., 2012; Rai et al., 2012; Bartels and Dinakar, 2013; Sreeshan et al., 2014; Joshi et al., 2015; Volkov, 2015; Muchate et al., 2016). It may be because of higher expression of key genes involved in the salt stress tolerance mechanism, or halophytic proteins are intrinsically more active than the corresponding glycophytic proteins (Anjum et al., 2012; Das and Strasser, 2013; Himabindu et al., 2016; Muchate et al., 2016).

Different genomic and transcriptomics efforts have been made to isolate salt responsive genes from some halophytes followed by their functional validation through transgenic approaches. The overexpression of several halophytic genes, under the control of a non-specific 35SCaMV promoter, have been claimed to enhance abiotic stress tolerance in the glycophytic recipients (Table 1). A number of crops have been transformed with halophytic genes for the improvement of salt tolerance. Most of these genes encode for Na^+^/H^+^ antiporters (vacuolar or plasma membrane), vacuolar pyrophosphatase, potassium transporters, ion channels, antioxidants, ROS scavengers, and proteins that involve in protective function and signal transduction. Additionally, some novel salt responsive genes were also cloned and characterized from halophytes like *S. brachiata* (Udawat et al., 2014, 2017; Singh et al., 2016).

**Table 1:** **Abiotic stress responsive genes of halophytic origin reported to enhance salt tolerance in glycophytic hosts**.

**Halophytes**	**Genes**	**Description**	**Recipient plants**	**References**
*Aeluropus littoralis*	*AlNHX1*	Vacuolar Na^+^/H^+^ antiporter	*Nicotiana tabacum*	Zhang et al., 2008
*Atriplex centralasiatica*	*AcBADH*	Synthesis of glycine betaine	*Nicotiana tabacum*	Yin et al., 2002
*Atriplex gmelini*	*AgNHX1*	Vacuolar Na^+^/H^+^ antiporter	*Oryza sativa*	Ohta et al., 2002
*Atriplex hortensis*	*AhBADH*	Synthesis of glycine betaine	*Tomato*	Jia et al., 2002
*Atriplex hortensis*	*AhProT1*	Proline transport	*Arabidopsis*	Shen et al., 2002
*Atriplex nummularia*	*AmCMO*	Enhanced glycine betaine synthesis	*Nicotiana tabacum*	Tabuchi et al., 2005
*Avicennia marina*	*AmMDHAR*	Ascorbate regeneration and ROS scavenging	*Nicotiana tabacum*	Kavitha et al., 2010
*Halostachys caspica*	*HcNHX1*	Vacuolar Na^+^/H^+^ antiporter	*Arabidopsis*	Guan et al., 2011
*Halostachys caspica*	*V-ATPase*	Vacuolar-H^+^-pyrophosphatase	*Arabidopsis*	Hu et al., 2012
*Kalidium foliatum*	*V-ATPase*	Vacuolar-H^+^-pyrophosphatase	*Arabidopsis*	Yao et al., 2012
*Salicornia brachiata*	*SbASR1*	Abscisic acid stress ripening-1	*Arachis hypogea*	Tiwari et al., 2015a
*Salicornia brachiata*	*SbGSTU*	Tau class glutathione transferases	*Nicotiana tabacum*	Jha et al., 2011
*Salicornia brachiata*	*SbMT-2*	Metallothionein: ROS scavenger	*Nicotiana tabacum*	Chaturvedi et al., 2014
*Salicornia brachiata*	*SbNHX1*	Vacuolar Na^+^/H^+^ antiporter	*Jatropha curcas*	Joshi et al., 2013
*Salicornia brachiata*	*SbNHX1*	Vacuolar Na^+^/H^+^ antiporter	*Ricinus communis*	Patel et al., 2015
*Salicornia brachiata*	*SbNHX1*	Vacuolar Na^+^/H^+^ antiporter	*Cuminum cyminum*	Pandey et al., 2016
*Salicornia brachiata*	*SbpAPX*	Peroxisomal ascorbate peroxidase	*Nicotiana tabacum*	Singh et al., 2014a
*Salicornia brachiata*	*SbpAPX*	Peroxisomal ascorbate peroxidase	*Arachis hypogea*	Singh et al., 2014b
*Salicornia brachiata*	*SbSDR1*	Salt and drought responsive gene	*Nicotiana tabacum*	Singh et al., 2016
*Salicornia brachiata*	*SbSRP*	Salt responsive protein encoding gene	*Nicotiana tabacum*	Udawat et al., 2017
*Salicornia brachiata*	*SbUSP*	Cytosolic universal stress protein	*Nicotiana tabacum*	Udawat et al., 2016
*Salicornia europaea*	*SeCMO*	Enhanced glycine betaine synthesis	*Nicotiana tabacum*	Wu et al., 2010
*Salsola soda*	*SsNHX1*	Vacuolar Na^+^/H^+^ antiporter	*Alfalfa*	Li et al., 2011
*Spartina alterniflora*	*SaVHAc1*	Vacuolar H + -ATPase subunit c1	*Oryza sativa*	Baisakh et al., 2012
*Suaeda corniculata*	*V-ATPase*	Vacuolar-H^+^-pyrophosphatase	*Arabidopsis*	Liu et al., 2011
*Suaeda liaotungensis*	*SlASR1*	Abscisic acid stress ripening	*Arabidopsis*	Hu et al., 2014
*Suaeda liaotungensis*	*SlBADH*	Synthesis of glycine betaine	*Zea mays*	Wu et al., 2008
*Suaeda liaotungensis*	*SlBADH*	Synthesis of glycine betaine	*Nicotiana tabacum*	Li et al., 2003a
*Suaeda liaotungensis*	*SlCMO*	Enhanced glycine betaine synthesis	*Nicotiana tabacum*	Li et al., 2003b
*Suaeda liaotungensis*	*SlNAC*	NAC transcription factor	*Arabidopsis*	Yang et al., 2014
*Suaeda salsa*	*SsCAX1*	Vacuolar H^+^/Ca^2+^ Transporter	*Arabidopsis*	Han et al., 2012
*Suaeda salsa*	*Ss.sAPX*	Stroma ascorbate peroxidase	*Arabidopsis*	Li et al., 2012
*Suaeda salsa*	*SsCHLAPX*	Chloroplastic ascorbate peroxidase	*Arabidopsis*	Pang et al., 2011
*Suaeda salsa*	*SsGST*	Glutathione *S*-transferase	*Oryza sativa*	Zhao and Zhang, 2006
*Suaeda salsa*	*SsPrxQ*	Chloroplast-located Peroxiredoxin Q	*Arabidopsis*	Jing et al., 2006
*Suaeda salsa*	*SsVP*	Vacuolar-H^+^-pyrophosphatase	*Arabidopsis*	Guo et al., 2006
*Tamarix androssowii*	*TaMnSOD*	Antioxidant: manganese superoxide dismutase	*Populus*	Wang et al., 2010
*Thellungiella halophila*	*ThNHX1*	Vacuolar Na^+^/H^+^ antiporter	*Arabidopsis*	Wu et al., 2009
*Thellungiella halophila*	*ThSOS1*	Salt overly sensitive gene	*Arabidopsis*	Oh et al., 2009
*Thellungiella halophila*	*TsVP*	H^+^-PPase gene	*Gossypium*	Lv et al., 2008
*Thellungiella halophila*	*TsVP*	H^+^-PPase gene	*Nicotiana tabacum*	Gao et al., 2006
*Thellungiella salsuginea*	*TsLEA1*	Late embryogenesis abundant (*LEA*)	*Arabidopsis*	Zhang et al., 2012
*Thellungiella salsuginea*	*TsTIP1*	Tonoplast AQP gene	*Arabidopsis*	Wang et al., 2014

A close relative of thoroughly explored glycophytic crucifer *A. thaliana, Thellungiella salsuginea*, which was earlier classified as *T. halophila* is a halophyte, exhibiting a high tolerance to salt and drought, considered as a potential model for abiotic stress tolerance studies by some researchers (Amtmann, 2009; Bartels and Dinakar, 2013). The genome sequence of *T. salsuginea* provides evidence about the genetic basis of abiotic stress defense mechanisms, and comparative genomics identified this plant as a gene resource for cation transporters, abscisic acid signaling genes, and other upregulated genes that showing a response to stressful environments (Wu et al., 2012). Furthermore, microarray analysis exhibited that only few genes were induced in *Thellungiella* compared to *Arabidopsis* under salt stress (Taji et al., 2004). Another study reveals that about 154 genes were differentially regulated in *Thellungiella* compared to *Arabidopsis* under varying stress (Wong et al., 2006).

Similarly, another halophytic relative of the model plant *Arabidopsis, Lepidium crassifolium* showed salt, osmotic and oxidative stresses tolerance. Random genes were transferred from *L. crassifolium* to *A. thaliana*, and it was observed that independent transgenic lines enhanced tolerance under several stress conditions (Rigó et al., 2016). Approximately 15% of functionally unknown genes were additionally expressed under salt stress compared to the non-stress conditions in *M. crystallinum* (Cushman and Bohnert, 2000; Kore-eda et al., 2004).

An extreme halophyte *S. brachiata* grows luxuriantly on salt marshes and also frequently encountered with different environmental stresses. Since, *S. brachiata* has unique opportunity to sustain adverse conditions and thus considered as a rich source of stress responsive genes and promoters (Jha et al., 2011; Chaturvedi et al., 2012; Singh et al., 2014a; Tiwari et al., 2014, 2016; Udawat et al., 2016). The salt responsive genes from *S. brachiata* have been utilized to develop salt stress tolerant transgenic crops such as jatropha, cumin, castor, and peanuts (Joshi et al., 2011; Singh et al., 2014b; Patel et al., 2015; Tiwari et al., 2015a; Jha et al., 2016; Pandey et al., 2016) using different genetic transformation methods (Singh et al., 2010; Joshi et al., 2012; Pandey et al., 2013; Tiwari et al., 2015b). Furthermore, *Salicornia* also owns unique oligosaccharides (Mishra et al., 2013), metabolites (Mishra et al., 2015), sulfur-rich seed-storage proteins (Jha et al., 2012) and thus considered as a functional food. Transcriptomics of *Porteresia coarctata*, a wild relative of rice showing high salinity and submergence tolerance revealed a total of 152,367 unique transcript sequences (Garg et al., 2014). A total of 15,158 genes, involved in salinity and submergence tolerance were identified to unravel key metabolic pathways. These genes can be explored further to understand and engineer salinity and submergence tolerance in rice (Garg et al., 2014).

### Promoters of salt-responsive halophytic genes: at a glance

A strong and well-regulated promoter is required for the engineering of crop plants to achieve the desired level of expression of a transgene. A comparative transcriptome analysis revealed that many stress-related genes constitutively expressed at higher level in *T. halophila* compared to their homologs of *A. thaliana* (Taji et al., 2004, 2010). This report suggests an efficient transcriptional regulatory network for stress responsive genes in halophytes. Recently, *cis*-regulatory elements of different stress responsive genes from some halophytes have been studied, and the presence of various stress-inducible motifs was observed (Tiwari et al., 2014, 2016). Yin et al. (2002) found that the promoter of *AcBADH* gene from *Atriplex centralasiatica* is strongly induced by salt stress and possesses two salt-responsive enhancer regions (located from −1,115 to −890 and −462 to −230) and one silencer region (located between −890 and −641).

The *SlBADH* gene promoter fragment (−300 bp only) from *Suaeda liaotungensis* showed about 6.3-fold expression under salt stress (400 mmol/l NaCl) compared to control (non-stressed) condition (Zhang et al., 2008). The *TsVP1* gene promoter from halophyte *T. halophila* contained 130 bp specific *cis*-acting element and showed a higher expression of GUS in transgenic *Arabidopsis* under salt stress (Sun et al., 2010). Similarly, an 897 bp promoter region of *SlPEAMT* gene (*S. liaotungensis*) showed an 18.6-fold increase in the GUS activity under NaCl stress (200 mmol/l) treatment (Li et al., 2016). These results suggest that even a small fragment of promoter can also contain essential *cis*-acting elements to regulate gene expression under stress. The promoters of *CMO* genes from *S. liaotungensis* and *Salicornia europaea* also possessed basic elements and demonstrated to be salt inducible (Li et al., 2007; Wu et al., 2011). Schaeffer et al. (1995) identified enhancer and silencer regions involved in the transcriptional activation of salt-responsive expression of CAM (Crassulacean Acid Metabolism) genes in the halophyte *M. crystallinum*.

An age-dependent, abiotic-stress-inducible, organ-specific, and tissue-specific promoter, *AlSAP* was reported from *Aeluropus littoralis* (Saad et al., 2011). Furthermore, *gusA* exhibited same expression level under the control of *AlSAP* gene promoter in transgenic rice as *AlSAP* transcript in *A. littoralis* (Ben-Saad et al., 2015). They also concluded that the regulatory regions of two orthologs *AlSAP* and *OsSAP9* (from rice) have a different specificity of regulation and stress induction in rice. Sun et al. (2010) found a 130 bp specific *cis*-acting element in the promoter region of vacuolar H^+^-pyrophosphatase from a halophyte *T. halophila* (*TsVP1)* which enhances the expression of GUS in transgenic *Arabidopsis* under salt stress. The *CBL1* gene promoter isolated from *Ammopiptanthus mongolicust* controlled the expression of the reporter gene under abiotic and biotic stress conditions (Guo et al., 2010). A model, proposed for transcriptional regulation of the *SbpAPX* gene (from *S. brachiata*) showed the presence of enhancer and repressor binding sites in the *cis*-regulatory elements along with stress-inducible motifs (Tiwari et al., 2014). Similarly, the *SbGSTU* promoter showed the presence of a number of abiotic stress responsive *cis*-regulatory motifs which regulate the expression of *GSTU* gene in *S. brachiata* (Tiwari et al., 2016). Therefore, based on different reports, halophytic promoters emerge as a promising candidate for engineering abiotic stress tolerance in crops for high-level expression of transgenes.

### Salt tolerant genes from halophytes and glycophytes: a comparative analysis

Among different strategies; Na^+^ efflux, compartmentalization of Na^+^ in vacuoles and prevention of Na^+^ influx are the most common, governed by antiporters and regulated by a multigene family (Rajendran et al., 2009; Kronzucker and Britto, 2011). A number of antiporters isolated from both glycophytes and halophytes are functionally characterized (Kronzucker and Britto, 2011; Sreeshan et al., 2014). The overexpression of glycophytic transporters encoding genes (*NHX, SOS, HKT, ATPase*, etc.), under the control of non-specific CaMV35S promoter, showed tolerance in the range of 150–250 mM NaCl, however their halophytic homologs may provide tolerance up to 400 mM NaCl (reviewed in Kronzucker and Britto, 2011; Sreeshan et al., 2014; Volkov, 2015). In several previous studies, the effects of overexpression of halophytic genes were commonly observed under salt stress treatments, however, negligible differences were observed between wild-type plants and the transgenic lines under control (unstressed) conditions (Jha et al., 2011; Joshi et al., 2012; Volkov, 2015; Tiwari et al., 2015a; Singh et al., 2016; Udawat et al., 2016, 2017).

The glycophytic *NHX* gene from *A. thaliana* was widely explored for developing salt tolerance in many crops including tomato, brassica, maize, wheat, etc. (Zhang et al., 2001; Xue et al., 2004; Yin et al., 2004). Even, other glycophytic *NHX1* genes such as *BnNHX1* (*Brassica napus*), *GhNHX1* (*Gossypium hirsutum*), and *HbNHX1* (*Hordeum brevisubulatum*) have demonstrated to produce salt tolerance in the model plant tobacco (Wang et al., 2004; Wu et al., 2004; Lü et al., 2005). Thus, the *NHX1* gene from halophyte and glycophyte both showed the salt tolerance activity, but there is a difference regarding salt tolerant intensity. The antiporter *Ag*NHX1 (from halophyte *Atriplex gmelini*) showed 75% amino-acid sequence similarity with *At*NHX1 (*A. thaliana*) and a higher salinity tolerance in *Oryza sativa* (Hamada et al., 2001; Ohta et al., 2002). Transgenic plants overexpressing *AgNHX1* (*A. gmelini*), *SaNHX1* (*Spartina anglica*) or *SsNHX1* (*Suaeda salsa*) gene show tolerance up to 300–400 mM NaCl compared to glycophytic counterparts (Ohta et al., 2002; Zhao et al., 2006; Lan et al., 2011). The overexpression of *SbNHX1* gene showed 200 mM salt tolerance in the model plant transgenic tobacco, but only 100 mM NaCl tolerance was observed in the transgenic jatropha and castor plants (Joshi et al., 2013; Patel et al., 2015).

Similar to *NHX* gene family, the overexpression of other halophytic genes such as *SbpAPX, SbUSP*, and *SbGSTU* showed better salinity tolerance (200–300 mM NaCl) in the transgenic plants compared to their glycophytic homologs (Jha et al., 2011; Singh et al., 2014a,b; Udawat et al., 2016). The transgenic *Arabidopsis* plants, overexpressing the *TIP1* gene from the halophyte *T. salsuginea* exhibited better salt tolerance compared to the same gene from glycophyte *Panax ginseng* (Peng et al., 2007). Similarly, *APX* and *GST* from rice showed lower tolerance up to 150–200 mM compared to the same genes (200–300 mM NaCl) from halophyte *S. brachiata* in the transgenic plants (Lu et al., 2007; Jha et al., 2011; Sharma et al., 2014; Singh et al., 2014a). Recently, it was reported that over-expression of a stress-associated protein gene (*AlSAP*) from *A. littoralis* improves different abiotic stress tolerance in tobacco, wheat, and rice (Ben-Saad et al., 2015). They also demonstrated that *AlSAP* transcripts are induced by multiple abiotic stresses, but the ortholog gene of rice *OsSAP9* is preferentially induced by cold and heat treatments.

A comparative transcript expression analysis revealed a higher expression of antiporter *SOS1* gene in *Thellungiella* species compared to *Arabidopsis* (Oh et al., 2010). Similarly, several genes such as *SOS2, NHX1*, and *HKT1* involved in Na^+^ excretion, compartmentation, and diffusion were also expressed at higher levels in *Thellungiella* compared to *Arabidopsis* (Taji et al., 2010). To compare the Na^+^ hypersensitivity response, *Arabidopsis* lines overexpressing either *AtHKT1* (*A. thaliana*) or *TsHKT1* (*T. salsuginea*) were analyzed and delayed root growth was observed in *AtHKT1* compared with those expressing *TsHKT1* (Ali et al., 2012). The shoot sensitivity was observed in transgenic lines expressing *AtHKT1*. They also demonstrated a strong salt-dependent up-regulation of *TsHKT1* but a strong repression of *AtHKT1* expression under salt stress (Ali et al., 2012).

Based on different reports, it may be concluded that halophytic genes are one of the promising candidates to be explored further for producing transgenic plants with a higher level of salt tolerance as compared to glycophytic counterpart genes. Further, halophytes also serve as valuable resources to discover novel abiotic stress responsive genes for improving stress tolerance of crop plants for sustainable agriculture in the saline affected areas.

## Perspective and conclusion

Halophytes are more tolerant to abiotic stress because of high differential regulation of the same basic set of stress-responsive genes present among all plants. Furthermore, halophytes exhibited higher expression of a large number of stress-inducible genes under the non-stress condition, suggesting constitutive expression of genes in halophytes. Since different halophytes use different mechanisms to respond the salt stress, a single species cannot be considered as a model species. However, the emergence of a halophyte species as a model plant for the molecular elucidation of corresponding abiotic stress tolerance will enlighten our understanding of the salinity tolerance mechanisms. Identification and isolation of novel salt responsive genes and promoters from different halophytes can be explored for the genetic engineering of crop plants for abiotic stress tolerance using transgenic approach.

## Author contributions

AM: Conceived the idea, collected literature and wrote the paper. BT: Helped in revisions. All authors approved this mini-review for the publication.

### Conflict of interest statement

The authors declare that the research was conducted in the absence of any commercial or financial relationships that could be construed as a potential conflict of interest.

## References

[B1] Ali Z. Park H. C. Ali A. Oh D. H. Aman R. Kropornicka A.. (2012). *TsHKT1*; 2, a *HKT1* homolog from the extremophile *Arabidopsis* relative *Thellungiella salsuginea*, shows K^+^ specificity in the presence of NaCl. Plant Physiol. 158, 1463–1474. 10.1104/pp.111.193110 22238420 PMC3291249

[B2] Amtmann A. (2009). Learning from evolution: *Thellungiella* generates new knowledge on essential and critical components of abiotic stress tolerance in plants. Mol. Plant 2, 3–12. 10.1093/mp/ssn094 19529830 PMC2639741

[B3] Anjum N. A. Gill S. S. Ahmad I. Tuteja N. Soni P. Pareek A. (2012). Understanding stress-responsive mechanisms in plants: an overview of transcriptomics and proteomics approaches, in Improving Crop Resistance to Abiotic Stress, Vols. 1, 2, eds Tuteja N. Gill S. S. Tiburcio A. F. Tuteja R. (Weinheim: Wiley-VCH Verlag GmbH and Co. KGaA), 337–355.

[B4] Aslam R. Bostan N. Maria M. Safdar W. (2011). A critical review on halophytes: salt tolerant plants. J. Med. Plants Res. 5, 7108–7118. 10.5897/JMPRX11.009

[B5] Baisakh N. RamanaRao M. V. Rajasekaran K. Subudhi P. Janda J. Galbraith D.. (2012). Enhanced salt stress tolerance of rice plants expressing a vacuolar H^+^-ATPase subunit c1 (*SaVHAc1*) gene from the halophyte grass *Spartina alterniflora* Löisel. Plant Biotechnol. J. 10, 453–464. 10.1111/j.1467-7652.2012.00678.x 22284568

[B6] Ball M. C. (1988). Ecophysiology of mangroves. Trees Struct. Funct. 2, 129–142. 10.1007/BF00196018

[B7] Bartels D. Dinakar C. (2013). Balancing salinity stress responses in halophytes and non-halophytes: a comparison between *Thellungiella* and *Arabidopsis thaliana*. Funct. Plant Biol. 40, 819–831. 10.1071/fp12299 32481153

[B8] Ben-Saad R. Meynard D. Ben-Romdhane W. Mieulet D. Verdeil J. L. Al-Doss A.. (2015). The promoter of the *AlSAP* gene from the halophyte grass *Aeluropus littoralis* directs a stress-inducible expression pattern in transgenic rice plants. Plant Cell Rep. 34, 1791–1806. 10.1007/s00299-015-1825-6 26123290

[B9] Braun-Blanquet J. (1932). Plant sociology: the Study of Plant Communities. Transl. by G. D. Fuller and H. S. Conard. New York; NY: McGraw-Hill Book Co.

[B10] Chaturvedi A. K. Mishra A. Tiwari V. Jha B. (2012). Cloning and transcript analysis of type 2 metallothionein gene (*SbMT-2*) from extreme halophyte *Salicornia brachiata* and its heterologous expression in E. coli. Gene 499, 280–287. 10.1016/j.gene.2012.03.001 22441126

[B11] Chaturvedi A. K. Patel M. K. Mishra A. Tiwari V. Jha B. (2014). The *SbMT-2* gene from a halophyte confers abiotic stress tolerance and modulates ROS scavenging in transgenic tobacco. PLoS ONE 9:e111379. 10.1371/journal.pone.0111379 25340650 PMC4207811

[B12] Colmer T. D. Flowers T. J. (2008). Flooding tolerance in halophytes. New Phytol. 179, 964–974. 10.1111/j.1469-8137.2008.02483.x 18482227

[B13] Cushman J. C. (2001). Osmoregulation in plants: implications for agriculture. Am. Zool. 41, 758–769. 10.1668/0003-1569(2001)041[0758:OIPIFA]2.0.CO;2

[B14] Cushman J. C. Bohnert H. J. (2000). Genomic approaches to plant stress tolerance. Curr. Opin. Plant Biol. 3, 117–124. 10.1016/S1369-5266(99)00052-7 10712956

[B15] Das A. B. Strasser R. J. (2013). Salinity-induced genes and molecular basis of salt-tolerant strategies in Mangroves, in Molecular Stress Physiology of Plants, eds Rout G. R. Das A. B. (Springer), 53–86.

[B16] FAO (2008). Land and Plant Nutrition Management Service. Rome: FAO.

[B17] FAO (2016). Food and Agriculture: Key to Achieving the 2030, Agenda for Sustainable Development. Job No. I5499, Food and Agriculture Organization of the United Nations, Rome, 23. Available online at: http://www.fao.org/3/a-i5499e.pdf

[B18] Flowers T. J. (1985). Physiology of halophytes. Plant Soil 89, 41–56. 10.1007/BF02182232

[B19] Flowers T. J. Colmer T. D. (2008). Salinity tolerance in halophytes. New Phytol. 179, 945–963. 10.1111/j.1469-8137.2008.02531.x 18565144

[B20] Flowers T. J. Colmer T. D. (2015). Plant salt tolerance: adaptations in halophytes. Ann. Bot. 115, 327–331. 10.1093/aob/mcu267 25844430 PMC4332615

[B21] Flowers T. J. Munns R. Colmer T. D. (2015). Sodium chloride toxicity and the cellular basis of salt tolerance in halophytes. Ann. Bot. 115, 419–431. 10.1093/aob/mcu217 25466549 PMC4332607

[B22] Gao F. Gao Q. Duan X. Yue G. Yang A. Zhang J. (2006). Cloning of an H^+^-PPase gene from *Thellungiella* halophila and its heterologous expression to improve tobacco salt tolerance. J. Exp. Bot. 57, 3259–3270. 10.1093/jxb/erl090 16940040

[B23] Garg R. Verma M. Agrawal S. Shankar R. Majee M. Jain M. (2014). Deep transcriptome sequencing of wild halophyte rice, *Porteresia coarctata*, provides novel insights into the salinity and submergence tolerance factors. DNA Res. 21, 69–84. 10.1093/dnares/dst042 24104396 PMC3925395

[B24] Grigore M. N. Ivanescu L. Toma C. (2014). Halophytes: An Integrative Anatomical Study. New York, NY: Springer.

[B25] Grigore M.-N. Toma C. (2010). A proposal for a new halophytes classification, based on integrative anatomy observations. Oltenia Stud. Comun. Ştiinţele Nat. (Oltenia J. Stud. Nat. Sci.) 26, 45–50. Available online at: http://biozoojournals.ro/oscsn/cont/26_1/B08-Grigore.pdf

[B26] Grigore M.-N. Toma C. Boşcaiu M. (2010). Dealing with halophytes: an old problem, the same continuous exciting challenge. An. Şt. Univ. Al. I. Cuza Iaşi 56, 21–32. Available online at: http://www.bio.uaic.ro/publicatii/anale_vegetala/issue/2010F1/03-2010F1.pdf

[B27] Grigore M. N. Villanueva M. Boscaiu M. Vicente O. (2012). Do halophytes really require salts for their growth and development? An experimental approach. Not. Sci. Biol. 4, 23–29. 10.15835/nsb427606

[B28] Guan B. Hu Y. Zeng Y. Wang Y. Zhang F. (2011). Molecular characterization and functional analysis of a vacuolar Na^+^/H^+^ antiporter gene (*HcNHX1*) from *Halostachys caspica*. Mol. Biol. Rep. 38, 1889–1899. 10.1007/s11033-010-0307-8 20886297

[B29] Guo L. Yu Y. Xia X. Yin W. (2010). Identification and functional characterisation of the promoter of the calcium sensor gene *CBL1* from the xerophyte *Ammopiptanthus mongolicus*. BMC Plant Biol. 10:18. 10.1186/1471-2229-10-18 20113489 PMC2844064

[B30] Guo S. Yin H. Zhang X. Zhao F. Li P. Chen S.. (2006). Molecular cloning and characterization of a vacuolar H^+^-pyrophos-phatase gene, *SsVP*, from the halophyte *Suaeda salsa* and its overexpression increases salt and drought tolerance of *Arabidopsis*. Plant Mol. Biol. 60, 41–50. 10.1007/s11103-005-2417-6 16463098

[B31] Hamada A. Shono M. Xia T. Ohta M. Hayashi Y. Tanaka A.. (2001). Isolation and characterization of a Na^+^/H^+^ antiporter gene from the halophyte *Atriplex gmelini*. Plant Mol. Biol. 46, 35–42. 10.1023/A:1010603222673 11437248

[B32] Han N. Lan W. He X. Shao Q. Wang B. Zhao X. (2012). Expression of a *Suaeda salsa* vacuolar H^+^/Ca^2+^ transporter gene in *Arabidopsis* contributes to physiological changes in salinity. Plant Mol. Biol. Rep. 30, 470–477. 10.1007/s11105-011-0353-y

[B33] Himabindu Y. Chakradhar T. Reddy M. C. Kanygin A. Redding K. E. Chandrasekhar T. (2016). Salt-tolerant genes from halophytes are potential key players of salt tolerance in glycophytes. Environ. Exp. Bot. 124, 39–63. 10.1016/j.envexpbot.2015.11.010

[B34] Hu Y. X. Yang X. Li X. L. Yu X. D. Li Q. L. (2014). The *SlASR* gene cloned from the extreme halophyte *Suaeda liaotungensis* K. enhances abiotic stress tolerance in transgenic *Arabidopsis thaliana*. Gene 549, 243–251. 10.1016/j.gene.2014.07.071 25088570

[B35] Hu Y. Z. Zeng Y. L. Guan B. Zhang F. C. (2012). Overexpression of a vacuolar H^+^-pyrophosphatase and a B subunit of H^+^-ATPase cloned from the halophyte *Halostachys caspica* improves salt tolerance in *Arabidopsis thaliana*. Plant Cell Tiss. Organ Cult. 108, 63–71. 10.1007/s11240-011-0013-9

[B36] Jha B. Mishra A. Chaturvedi A. K. (2016). Engineering stress tolerance in peanut (*Arachis hypogaea* L.), in Genetically Modified Organisms (GMO) Foods: Production, Regulation and Public Health, eds Watson R. Preedy V. R. (Philadelphia, PA: Elsevier), 305–311. 10.1016/B978-0-12-802259-7.00027-0

[B37] Jha B. Sharma A. Mishra A. (2011). Expression of *SbGSTU* (tau class glutathione S-transferase) gene isolated from *Salicornia brachiata* in tobacco for salt tolerance. Mol. Biol. Rep. 38, 4823–4832. 10.1007/s11033-010-0625-x 21136169

[B38] Jha B. Singh N. P. Mishra A. (2012). Proteome profiling of seed storage proteins reveals the nutritional potential of *Salicornia brachiata* Roxb., an extreme halophyte. J. Agric. Food Chem. 60, 4320–4326. 10.1021/jf203632v 22494338

[B39] Jia G. X. Zhu Z. Q. Chang F. Q. Li Y. X. (2002). Transformation of tomato with the BADH gene from *Atriplex* improves salt tolerance. Plant Cell Rep. 21, 141–146. 10.1007/s00299-002-0489-1

[B40] Jing L. W. Chen S. H. Guo X. L. Zhang H. Zhao Y. X. (2006). Overexpression of a chloroplast-located peroxiredoxin Q gene, *SsPrxQ*, increases the salt and low-temperature tolerance of *Arabidopsis*. J. Integr. Plant Biol. 48, 1244–1249. 10.1111/j.1744-7909.2006.00357.x

[B41] Jithesh M. N. Prashanth S. R. Sivaprakash K. R. Parida A. K. (2006). Antioxidative response mechanisms in halophytes: their role in stress defence. J. Genet. 85, 237–254. 10.1007/bf02935340 17406103

[B42] Joshi M. Jha A. Mishra A. Jha B. (2013). Developing transgenic *Jatropha* using the *SbNHX1* gene from an extreme halophyte for cultivation in saline wasteland. PLoS ONE 8:e71136. 10.1371/journal.pone.0071136 23940703 PMC3733712

[B43] Joshi M. Mishra A. Jha B. (2011). Efficient genetic transformation of *Jatropha curcas* L. by microprojectile bombardment using embryo axes. Ind. Crops Prod. 33, 67–77. 10.1016/j.indcrop.2010.09.002

[B44] Joshi M. Mishra A. Jha B. (2012). NaCl plays a key role for *in vitro* micropropagation of *Salicornia brachiata*, an extreme halophyte. Ind. Crops Prod. 35, 313–316. 10.1016/j.indcrop.2011.06.024

[B45] Joshi R. Mangu V. R. Bedre R. Sanchez L. Pilcher W. Zandkarimi H. (2015). Salt adaptation mechanisms of halophytes: improvement of salt tolerance in crop plants, in Elucidation of Abiotic Stress Signaling in Plants, ed Pandey G. K. (New York, NY: Springer), 243–279.

[B46] Kavitha K. George S. Venkataraman G. Parida A. (2010). A salt-inducible chloroplastic monodehydroascorbate reductase from halophyte *Avicennia marina* confers salt stress tolerance on transgenic plants. Biochimie 92, 1321–1329. 10.1016/j.biochi.2010.06.009 20600571

[B47] Khan M. S. Khan M. A. Ahmad D. (2016). Assessing utilization and environmental risks of important genes in plant abiotic stress tolerance. Front. Plant Sci. 7:792. 10.3389/fpls.2016.00792 27446095 PMC4919908

[B48] Kore-eda S. Cushman M. A. Akselrod I. Bufford D. Fredrickson M. Clark E.. (2004). Transcript profiling of salinity stress responses by large-scale expressed sequence tag analysis in *Mesembryanthemum crystallinum*. Gene 341, 83–92. 10.1016/j.gene.2004.06.037 15474291

[B49] Kronzucker H. J. Britto D. T. (2011). Sodium transport in plants: a critical review. New Phytol. 189, 54–81. 10.1111/j.1469-8137.2010.03540.x 21118256

[B50] Lan T. Duan Y. Wang B. Zhou Y. Wu W. (2011). Molecular cloning and functional characterization of a Na^+^/H^+^ antiporter gene from halophyte *Spartina anglica*. Turk. J. Agric. For. 35, 535–543. 10.3906/tar-1003-2

[B51] Li K. Pang C. H. Ding F. Sui N. Feng Z. T. Wang B. S. (2012). Overexpression of *Suaeda salsa* stroma ascorbate peroxidase in *Arabidopsis* chloroplasts enhances salt tolerance of plants. S. Afr. J. Bot. 78, 235–245. 10.1016/j.sajb.2011.09.006

[B52] Li Q. Yin H. Li D. Zhu H. Zhang Y. Zhu W. (2007). Isolation and characterization of CMO gene promoter from halophyte *Suaeda liaotungensis* K. J. Genet. Genomics 34, 355–361. 10.1016/S1673-8527(07)60038-1 17498634

[B53] Li Q.-L. Gao X.-R. Yu X.-H. Wang X.-Z. An L.-J. (2003a). Molecular cloning and characterization of betaine aldehyde dehydrogenase gene from *Suaeda liaotungensis* and its use in improved tolerance to salinity in transgenic tobacco. Biotechnol. Lett. 25, 1431–1436. 10.1023/A:1025003628446 14514045

[B54] Li Q.-L. Liu D.-W. Gao X.-R. Su Q. An L.-J. (2003b). Cloning of cDNA encoding choline monooxygenase from *Suaeda liaotungensis* and salt tolerance of transgenic tobacco. Acta Bot. Sin. 45, 242–247. Available online at: http://www.jipb.net/pubsoft/content/2/2318/X010047(PS2).pdf

[B55] Li Q.-L. Xie J.-H. Ma X.-Q. Li D. (2016). Molecular cloning of Phosphoethanolamine *N*-methyltransferase (PEAMT) gene and its promoter from the halophyte *Suaeda liaotungensis* and their response to salt stress. Acta Physiol. Plant. 38, 39 10.1007/s11738-016-2063-4

[B56] Li W. Wang D. Jin T. Chang Q. Yin D. Xu S. (2011). The vacuolar Na^+^/H^+^ antiporter gene *SsNHX1* from the halophyte *Salsola soda* confers salt tolerance in transgenic alfalfa (*Medicago sativa* L.). Plant Mol. Biol. Rep. 29, 278–290. 10.1007/s11105-010-0224-y

[B57] Liu L. Wang Y. Wang N. Dong Y. Y. Fan X. D. Liu X. M.. (2011). Cloning of a vacuolar H^+^-pyrophosphatase gene from the halophyte *Suaeda corniculata* whose heterologous overexpression improves salt, saline-alkali and drought tolerance in *Arabidopsis*. J. Integr. Plant Biol. 53, 731–742. 10.1111/j.1744-7909.2011.01066.x 21762382

[B58] Lokhande V. H. Suprasanna P. (2012). Prospects of halophytes in understanding and managing abiotic stress tolerance, in Environmental Adaptations and Stress Tolerance of Plants in the Era of Climate Change, eds Ahmad P. Prasad M. N. V. (New York, NY: Springer), 29–56.

[B59] Lovelock C. E. Ball M. C. (2002). Influence of salinity on photosynthesis of halophytes, in Salinity: Environment-Plants-Molecules, eds Läuchli A. Lüttge U. (Netherlands: Springer), 315–339.

[B60] Lü S. Y. Jing Y. X. Shen S. H. Zhao H. Y. Ma L. Q. Zhou X. J. (2005). Antiporter gene from *Hordum brevisubulatum* (Trin.) link and its overexpression in transgenic tobaccos. J. Integr. Plant Biol. 47, 343–349. 10.1111/j.1744-7909.2005.00027.x

[B61] Lu Z. Liu D. Liu S. (2007). Two rice cytosolic ascorbate peroxidases differentially improve salt tolerance in transgenic *Arabidopsis*. Plant Cell Rep. 26, 1909–1917. 10.1007/s00299-007-0395-7 17571267

[B62] Lv S. Zhang K. Gao Q. Lian L. Song Y. Zhang J. (2008). Overexpression of an H^+^-PPase gene from *Thellungiella halophila* in cotton enhances salt tolerance and improves growth and photosynthetic performance. Plant Cell Physiol. 49, 1150–1164. 10.1093/pcp/pcn090 18550626

[B63] Megdiche W. Passaquet C. Zourrig W. Fodil Y. Z. Abdelly C. (2009). Molecular cloning and characterization of novel cystatin gene in leaves *Cakile maritima* halophyte. J. Plant Physiol. 166, 739–749. 10.1016/j.jplph.2008.09.012 19042057

[B64] Mishra A. Joshi M. Jha B. (2013). Oligosaccharide mass profiling of nutritionally important *Salicornia brachiata*, an extreme halophyte. Carbohyd. Polym. 92, 1942–1945. 10.1016/j.carbpol.2012.11.055 23399241

[B65] Mishra A. Patel M. K. Jha B. (2015). Non targeted metabolomics and scavenging activity of reactive oxygen species reveal the potential of *Salicornia brachiata* as a functional food. J. Funct. Foods 13, 21–31. 10.1016/j.jff.2014.12.027

[B66] Muchate N. S. Nikalje G. C. Rajurkar N. S. Suprasanna P. Nikam T. D. (2016). Plant salt stress: adaptive responses, tolerance mechanism and bioengineering for salt tolerance. Bot. Rev. 82, 371–406. 10.1007/s12229-016-9173-y

[B67] Oh D. H. Dassanayake M. Haas J. S. Kropornika A. Wright C. d'Urzo M. P. (2010). Genome structures and halophyte-specific gene expression of the extremophile *Thellungiella parvula* in comparison with *Thellungiella salsuginea* (*Thellungiella halophila*) and *Arabidopsis*. Plant Physiol. 154, 1040–1052. 10.1104/pp.110.163923 20833729 PMC2971586

[B68] Oh D. H. Leidi E. Zhang Q. Hwang S. M. Li Y. Quintero F. J.. (2009). Loss of halophytism by interference with *SOS1* expression. Plant Physiol. 151, 210–222. 10.1104/pp.109.137802 19571313 PMC2735974

[B69] Ohta M. Hayashi Y. Nakashima A. Hamada A. Tanaka A. Nakamura T.. (2002). Introduction of a Na^+^/H^+^ antiporter gene from *Atriplex gmelini* confers salt tolerance to rice. FEBS Lett. 532, 279–282. 10.1016/S0014-5793(02)03679-7 12482579

[B70] Pandey S. Mishra A. Patel M. K. Jha B. (2013). An efficient method for *Agrobacterium*-mediated genetic transformation and plant regeneration in cumin (*Cuminum cyminum* L.). App. Biochem. Biotechnol. 171, 1–9. 10.1007/s12010-013-0349-1 23813408

[B71] Pandey S. Patel M. K. Mishra A. Jha B. (2016). *In planta* transformed cumin (*Cuminum cyminum* L.) plants, overexpressing the *SbNHX1* gene showed enhanced salt endurance. PLoS ONE 11:e0159349. 10.1371/journal.pone.0159349 27411057 PMC4943630

[B72] Pang C. H. Li K. Wang B. (2011). Overexpression of *SsCHLAPXs* confers protection against oxidative stress induced by high light in transgenic *Arabidopsis* thaliana. Physiol. Plant. 143, 355–366. 10.1111/j.1399-3054.2011.01515.x 21895668

[B73] Patel M. K. Joshi M. Mishra A. Jha B. (2015). Ectopic expression of *SbNHX1* gene in transgenic castor (*Ricinus communis* L.) enhances salt stress by modulating physiological process. Plant Cell Tiss. Organ Cult. 122, 477–490. 10.1007/s11240-015-0785-4

[B74] Patel M. K. Mishra A. Jha B. (2016). Untargeted metabolomics of halophytes, in Marine Omics: Principles and Applications, ed Kim S. (Boca Raton, FL: CRC Press), 309–325. 10.1201/9781315372303-18

[B75] Peng Y. Lin W. Cai W. Arora R. (2007). Overexpression of a *Panax ginseng* tonoplast aquaporin alters salt tolerance, drought tolerance and cold acclimation ability in transgenic *Arabidopsis* plants. Planta 226, 729–740. 10.1007/s00425-007-0520-4 17443343

[B76] Polunin N. (1960). Introduction to Plant Geography. New York, NY: McGraw-Hill Book Company, Inc.

[B77] Rai V. Tuteja N. Takabe T. (2012). Transporters and abiotic stress tolerance in plants, in Improving Crop Resistance to Abiotic Stress, Vols. 1, 2, eds Tuteja N. Gill S. S. Tiburcio A. F. Tuteja R. (Weinheim: Wiley-VCH Verlag GmbH & Co. KGaA), 507–522.

[B78] Rajalakshmi S. Parida A. (2012). Halophytes as a source of genes for abiotic stress tolerance. Plant Biochem. Biot. 21, 63–67. 10.1007/s13562-012-0146-x

[B79] Rajendran K. Tester M. Roy S. J. (2009). Quantifying the three main components of salinity tolerance in cereals. Plant Cell Environ. 32, 237–249. 10.1111/j.1365-3040.2008.01916.x 19054352

[B80] Rigó G. Valkai I. Faragó D. Kiss E. Van Houdt S. Van de Steene N.. (2016). Gene mining in halophytes: functional identification of stress tolerance genes in *Lepidium crassifolium*. Plant Cell Environ. 39, 2074–2084. 10.1111/pce.12768 27343166

[B81] Rozema J. (1995). Biology of halophytes, in Halophytes and Biosaline Agriculture, eds Choukr-AllAh R. Malcolm C. V. Hamdy A. (New York, NY: Marcel Dekker Inc), 17–30.

[B82] Rozema J. Muscolo A. Flowers T. (2013). Sustainable cultivation and exploitation of halophyte crops in a salinising world. Environ. Exp. Bot. 92, 1–3. 10.1016/j.envexpbot.2013.02.001

[B83] Rozema J. Schat H. (2013). Salt tolerance of halophytes, research questions reviewed in the perspective of saline agriculture. Environ. Exp. Bot. 92, 83–95. 10.1016/j.envexpbot.2012.08.004

[B84] Rozema J. Van Diggelen J. (1991). A comparative study of growth and photosynthesis of four halophytes in response to salinity. Acta Oecol. 12, 673–681.

[B85] Saad R. B. Romdhan W. B. Zouari N. Azaza J. Mieulet D. Verdeil J. L.. (2011). Promoter of the *AlSAP* gene from the halophyte grass *Aeluropus littoralis* directs developmental-regulated, stress-inducible, and organ-specific gene expression in transgenic tobacco. Transgenic Res. 20, 1003–1018. 10.1007/s11248-010-9474-6 21188636

[B86] Sahu B. B. Shaw B. P. (2009). Isolation, identification and expression analysis of salt-induced genes in *Suaeda maritima*, a natural halophyte, using PCR-based suppression subtractive hybridization. BMC Plant Biol. 9:69. 10.1186/1471-2229-9-69 19497134 PMC2702304

[B87] Sanders D. (2000). The salty tale of *Arabidopsis*. Curr. Biol. 10, R486–R488. 10.1016/S0960-9822(00)00554-6 10898972

[B88] Schaeffer H. J. Forsthoefel N. R. Cushman J. C. (1995). Identification of enhancer and silencer regions involved in salt-responsive expression of crassulacean acid metabolism (CAM) genes in the facultative halophyte *Mesembryanthemum crystallinum*. Plant Mol. Biol. 28, 205–218. 10.1007/BF00020241 7599307

[B89] Shabala S. (2013). Learning from halophytes: Physiological basis and strategies to improve abiotic stress tolerance in crops. Ann. Bot. 112, 1209–1221. 10.1093/aob/mct205 24085482 PMC3806534

[B90] Shabala S. Bose J. Hedrich R. (2014). Salt bladders: do they matter? Trends. Plant Sci. 19, 687–691. 10.1016/j.tplants.2014.09.001 25361704

[B91] Sharma R. Sahoo A. Devendran R. Jain M. (2014). Over-expression of a rice tau class glutathione s-transferase gene improves tolerance to salinity and oxidative stresses in *Arabidopsis*. PloS ONE 9:e92900. 10.1371/journal.pone.0092900 24663444 PMC3963979

[B92] Sharma R. Wungrampha S. Singh V. Pareek A. Sharma M. K. (2016). Halophytes as bioenergy crops. Front. Plant Sci. 7:1372. 10.3389/fpls.2016.01372 27679645 PMC5020079

[B93] Shen Y.-G. Zhang W.-K. Yan D.-Q. Du B.-X. Zhang J.-S. Chen S.-Y. (2002). Overexpression of proline transporter gene isolated from halophyte confers salt tolerance in *Arabidopsis*. Acta Bot. Sin. 44, 956–962. Available online at: http://www.jipb.net/pubsoft/content/2/2313/X010587(PS2).pdf

[B94] Singh N. Mishra A. Jha B. (2014a). Over-expression of the peroxisomal ascorbate peroxidase (*SbpAPX*) gene cloned from halophyte *Salicornia brachiata* confers salt and drought stress tolerance in transgenic tobacco. Mar. Biotechnol. 16, 321–332. 10.1007/s10126-013-9548-6 24197564

[B95] Singh N. Mishra A. Jha B. (2014b). Ectopic over-expression of peroxisomal ascorbate peroxidase (*SbpAPX*) gene confers salt stress tolerance in transgenic peanut (*Arachis hypogaea*). Gene 547, 119–125. 10.1016/j.gene.2014.06.037 24954532

[B96] Singh N. Mishra A. Joshi M. Jha B. (2010). Microprojectile bombardment mediated genetic transformation of embryo axis and plant regeneration in cumin (*Cuminum cyminum* L.). Plant Cell Tiss. Organ Cult. 103, 1–6. 10.1007/s11240-010-9746-0

[B97] Singh V. K. Mishra A. Haque I. Jha B. (2016). A novel transcription factor-like gene *SbSDR1* acts as a molecular switch and confers salt and osmotic endurance to transgenic tobacco. Sci. Rep. 6:31686 10.1038/srep31686 27550641 PMC4994045

[B98] Slama I. Abdelly C. Bouchereau A. Flowers T. Savoure A. (2015). Diversity, distribution and roles of osmoprotective compounds accumulated in halophytes under abiotic stress. Ann. Bot. 115, 433–447. 10.1093/aob/mcu239 25564467 PMC4332610

[B99] Sreeshan A. Meera S. P. Augustine A. (2014). A review on transporters in salt tolerant mangroves. Trees 28, 957–960. 10.1007/s00468-014-1034-x

[B100] Sun Q. Gao F. Zhao L. Li K. Zhang J. (2010). Identification of a new 130 bp cis-acting element in the *TsVP1* promoter involved in the salt stress response from *Thellungiella halophila*. BMC Plant Biol. 10:90. 10.1186/1471-2229-10-90 20482790 PMC3017807

[B101] Tabuchi T. Kawaguchi Y. Azuma T. Nanmori T. Yasuda T. (2005). Similar regulation patterns of choline monooxygenase, phosphoethanolamine N-methyltransferase and S-adenosyl-L-methionine synthetase in leaves of the halophyte *Atriplex nummularia* L. Plant Cell Physiol. 46, 505–513. 10.1093/pcp/pci050 15695433

[B102] Taji T. Komatsu K. Katori T. Kawasaki Y. Sakata Y. Tanaka S.. (2010). Comparative genomic analysis of 1047 completely sequenced cDNAs from an *Arabidopsis*-related model halophyte, *Thellungiella halophila*. BMC Plant Biol. 10:261. 10.1186/1471-2229-10-261 21106055 PMC3017837

[B103] Taji T. Seki M. Satou M. Sakurai T. Kobayashi M. Ishiyama K.. (2004). Comparative genomics in salt tolerance between *Arabidopsis* and Arabidopsis-related halophyte salt cress using *Arabidopsis* microarray. Plant Physiol. 135, 1697–1709. 10.1104/pp.104.039909 15247402 PMC519083

[B104] Tiwari V. Chaturvedi A. K. Mishra A. Jha B. (2014). The transcriptional regulatory mechanism of the peroxisomal ascorbate peroxidase (*pAPX*) gene cloned from an extreme halophyte, *Salicornia brachiata*. Plant Cell Physiol. 55, 1774–1471. 10.1093/pcp/pct172 24285755

[B105] Tiwari V. Chaturvedi A. K. Mishra A. Jha B. (2015a). Introgression of the *SbASR*−1 gene cloned from a halophyte *Salicornia brachiata* enhances salinity and drought endurance in transgenic groundnut (*Arachis hypogaea*) and acts as a transcription factor. PLoS ONE 10:e0135541 10.1371/journal.pone.0131567 26158616 PMC4497679

[B106] Tiwari V. Chaturvedi A. K. Mishra A. Jha B. (2015b). An efficient method of *Agrobacterium* mediated genetic transformation and regeneration in local Indian cultivar of Groundnut (*Arachis hypogaea*) using grafting. App. Biochem. Biotechnol. 175, 436–453. 10.1007/s12010-014-1286-3 25308617

[B107] Tiwari V. Patel M. K. Chaturvedi A. K. Mishra A. Jha B. (2016). Functional characterization of the tau class Glutathione-S-Transferases gene (*SbGSTU*) promoter of *Salicornia brachiata* under salinity and osmotic Stress. PLoS ONE 11:e0148494. 10.1371/journal.pone.0148494 26885663 PMC4757536

[B108] Udawat P. Jha R. K. Mishra A. Jha B. (2017). Overexpression of a plasma membrane-localized *Sb*SRP-like protein enhances salinity and osmotic stress tolerance in transgenic tobacco. Front. Plant Sci. 8:582. 10.3389/fpls.2017.00582 28473839 PMC5397517

[B109] Udawat P. Jha R. K. Sinha D. Mishra A. Jha B. (2016). Overexpression of a cytosolic abiotic stress responsive universal stress protein (*Sb*USP) mitigates salt and osmotic stress in transgenic tobacco plants. Front. Plant Sci. 7:518. 10.3389/fpls.2016.00518 27148338 PMC4838607

[B110] Udawat P. Mishra A. Jha B. (2014). Heterologous expression of an uncharacterized universal stress protein gene (*SbUSP*) from the extreme halophyte, *Salicornia brachiata*, which confers salt and osmotic tolerance to *E. coli*. Gene 536, 163–170. 10.1016/j.gene.2013.11.020 24291028

[B111] Ventura Y. Eshel A. Pasternak D. Sagi M. (2015). The development of halophyte-based agriculture: past and present. Ann. Bot. 115, 529–540. 10.1093/aob/mcu173 25122652 PMC4332600

[B112] Volkov V. (2015). Salinity tolerance in plants. Quantitative approach to ion transport starting from halophytes and stepping to genetic and protein engineering for manipulating ion fluxes. Front. Plant Sci. 6:873. 10.3389/fpls.2015.00873 26579140 PMC4621421

[B113] Waisel Y. (1972). Biology of Halophytes (New York, NY: Academic press).

[B114] Wang L. L. Chen A. P. Zhong N. Q. Liu N. Wu X. M. Wang F.. (2014). The *Thellungiella salsuginea* tonoplast aquaporin *TsTIP1*; 2 functions in protection against multiple abiotic stresses. Plant Cell Physiol. 55, 148–161. 10.1093/pcp/pct166 24214268 PMC3894706

[B115] Wang Y. C. Qu G. Z. Li H. Y. Wu Y. J. Wang C. Liu G. F.. (2010). Enhanced salt tolerance of transgenic poplar plants expressing a manganese superoxide dismutase from *Tamarix androssowii*. Mol. Biol. Rep. 37, 1119–1124. 10.1007/s11033-009-9884-9 19830589

[B116] Wang Z. Li P. Fredricksen M. Gong Z. Kim C. S. Zhang C. (2004). Expressed sequence tags from *Thellungiella halophila*, a new model to study plant salt-tolerance. Plant Sci. 166, 609–616. 10.1016/j.plantsci.2003.10.030

[B117] Wong C. E. Li Y. Labbe A. Guevara D. Nuin P. Whitty B.. (2006). Transcriptional profiling implicates novel interactions between abiotic stress and hormonal responses in *Thellungiella*, a close relative of *Arabidopsis*. Plant Physiol. 140, 1437–1450. 10.1104/pp.105.070508 16500996 PMC1435811

[B118] Wu C. A. Yang G. D. Meng Q. W. Zheng C. C. (2004). The cotton *GhNHX1* gene encoding a novel putative tonoplast Na^+^/H^+^ antiporter plays an important role in salt stress. Plant Cell Physiol. 45, 600–607. 10.1093/pcp/pch071 15169942

[B119] Wu C. Gao X. Kong X. Zhao Y. Zhang H. (2009). Molecular cloning and functional analysis of a Na^+^/H^+^ antiporter gene *ThNHX1* from a halophytic plant *Thellungiella halophila*. Plant Mol. Biol. Rep. 27, 1–12. 10.1007/s11105-008-0048-1

[B120] Wu H. J. Zhang Z. Wang J. Y. Oh D. H. Dassanayake M. Liu B.. (2012). Insights into salt tolerance from the genome of *Thellungiella salsuginea*. Proc. Natl. Acad. Sci. U.S.A. 109, 12219–12224. 10.1073/pnas.1209954109 22778405 PMC3409768

[B121] Wu S. Su Q. An L. J. (2010). Isolation of choline monooxygenase (CMO) gene from *Salicornia europaea* and enhanced salt tolerance of transgenic tobacco with CMO genes. Ind. J. Biochem. Biophys. 47, 298–305. 21280567

[B122] Wu S. Su Q. An L. Ma S. (2011). A choline monooxygenase gene promoter from *Salicornia europaea* increases expression of the-glucuronidase gene under abiotic stresses in tobacco (*Nicotiana tabacum* L.). Indian J Biochem. Biophys. 48, 170–174. 21793308

[B123] Wu W. Su Q. Xia X. Y. Wang Y. Luan Y. S. An L. J. (2008). The *Suaeda liaotungensis* kitag betaine aldehyde dehydrogenase gene improves salt tolerance of transgenic maize mediated with minimum linear length of DNA fragment. Euphytica 159, 17–25. 10.1007/s10681-007-9451-1

[B124] Xu C. Tang X. Shao H. Wang H. (2016). Salinity tolerance mechanism of economic halophytes from physiological to molecular hierarchy for improving food quality. Curr. Genomics 17, 207–214. 10.2174/1389202917666160202215548 27252587 PMC4869007

[B125] Xue Z. Y. Zhi D. Y. Xue G. P. Zhang H. Zhao Y. X. Xia G. M. (2004). Enhanced salt tolerance of transgenic wheat (*Tritivum aestivum* L.) expressing a vacuolar Na^+^/H^+^ antiporter gene with improved grain yields in saline soils in the field and a reduced level of leaf Na^+^. Plant Sci. 167, 849–859. 10.1016/j.plantsci.2004.05.034

[B126] Yang X. Hu Y. X. Li X. L. Yu X. D. Li Q. L. (2014). Molecular characterization and functional analysis of *SlNAC2* in *Suaeda liaotungensis* K. Gene 543, 190–197. 10.1016/j.gene.2014.04.025 24747017

[B127] Yao M. Zeng Y. Liu L. Huang Y. Zhao E. Zhang F. (2012). Overexpression of the halophyte *Kalidium foliatum* H^+^-pyrophosphatase gene confers salt and drought tolerance in *Arabidopsis thaliana*. Mol. Biol. Rep. 39, 7989–7996. 10.1007/s11033-012-1645-5 22539184

[B128] Yin X.-Y. Yang A.-F. Zhang K.-W. Zhang J.-R. (2004). Production and analysis of transgenic maize with improved salt tolerance by the introduction of *AtNHX1* gene. Acta Bot. Sin-Engl. 46, 854–861. Available online at: http://www.jipb.net/pubsoft/content/2/3522/x030020.pdf

[B129] Yin X. Zhao Y. Luo D. Zhang H. (2002). Isolating the promoter of a stress-induced gene encoding betaine aldehyde dehydrogenase from the halophyte *Atriplex centralasiatica* Iljin. BBA Gene Struct. Expr. 1577, 452–456. 10.1016/S0167-4781(02)00495-5 12359336

[B130] Zhang G. H. Su Q. An L. J. Wu S. (2008). Characterization and expression of a vacuolar Na^+^/H^+^ antiporter gene from the monocot halophyte *Aeluropus littoralis*. Plant Physiol. Biochem. 46, 117–126. 10.1016/j.plaphy.2007.10.022 18061467

[B131] Zhang H. X. Hodson J. N. Williams J. P. Blumwald E. (2001). Engineering salt-tolerant *Brassica* plants: characterization of yield and seed oil quality in transgenic plants with increased vacuolar sodium accumulation. Proc. Natl. Acad. Sci. U.S.A. 98, 12832–12836. 10.1073/pnas.231476498 11606781 PMC60139

[B132] Zhang Y. Li Y. Lai J. Zhang H. Liu Y. Liang L.. (2012). Ectopic expression of a LEA protein gene *TsLEA1* from *Thellungiella salsuginea* confers salt-tolerance in yeast and *Arabidopsis*. Mol. Biol. Rep. 39, 4627–4633. 10.1007/s11033-011-1254-8 21947846

[B133] Zhao F. Y. Zhang X. J. Li P. H. Zhao Y. X. Zhang H. (2006). Co-expression of the *Suaeda salsa* SsNHX1 and *Arabidopsis AVP1* confer greater salt tolerance to transgenic rice than the single *SsNHX1*. Mol. Breed. 17, 341–353. 10.1007/s11032-006-9005-6

[B134] Zhao F. Zhang H. (2006). Salt and paraquat stress tolerance results from co-expression of the *Suaeda salsa* glutathione S-transferase and catalase in transgenic rice. Plant cell Tiss. Organ cult. 86, 349–358. 10.1007/s11240-006-9133-z

[B135] Zhu J. K. (2000). Genetic analysis of plant salt tolerance using *Arabidopsis*. Plant Physiol. 124, 941–948. 10.1104/pp.124.3.941 11080272 PMC1539290

[B136] Zhu J. K. (2001). Plant salt tolerance. Trends Plant Sci. 6, 66–71. 10.1016/S1360-1385(00)01838-0 11173290

